# Factors that Promote Resilience in Homeless Children and Adolescents in Ghana: A Qualitative Study

**DOI:** 10.3390/bs9060064

**Published:** 2019-06-18

**Authors:** Kwaku Oppong Asante

**Affiliations:** Department of Psychology, University of Ghana, Legon, Accra, Ghana; koppongasante@ug.edu.gh or kwappong@gmail.com

**Keywords:** resilience, homeless youth, protective factors, survival, qualitative

## Abstract

Several studies conducted on street youth have focused on causes of homelessness, their engagement in risky sexual behaviours and the prevalence of STIs, including HIV/AIDS. Although homeless youth are considered resilient, sparse literature exists on factors that promote resilience in this vulnerable group. Using a qualitative approach, semi-structured interviews were conducted with 16 purposively selected homeless children and youth (with a mean age of 14 years) from the Central Business District of Accra, Ghana. Thematic analysis was used to analyse the data. Findings showed that a strong religious belief, sense of humour, engagement in meaningful social interactive activities, reciprocal friendship, adherence to cultural norms and support from community-based organizations were identified as factors that help homeless youth cope with the multiple challenges of street life. Strengthening such protective factors could help ameliorate the impact of adverse conditions of these street youth.

## 1. Introduction

Homeless youth are considered one of the most vulnerable groups in society as they are exposed to greater risks compared to other young adults. According to UNICEF [1], hundreds of millions of children are growing up on urban streets around the world. They, however, acknowledged that the exact number of street children is impossible to quantify, but the figures almost certainly run into millions across the world. In Ghana, the population of homeless youth is growing in cities such as Accra. Headcounts of street children ranged from 35,000 in 2009 to 90,000 in 2013 [2,3]. The main reasons identified for this high rate of homelessness include poverty, disintegrated families and divorce, and the quest for freedom from parental control [4,5,6,7].

In urban centres like Accra, street children work mainly as porters and sales workers and sometimes as child commercial sex workers. These activities expose them to great risks such as violence, sexual abuse, serious physical and psychological harm and sexually transmitted infections including HIV and AIDS [5,8,9]. What makes the already difficult situation worse is that these homeless youths in general are seen as a societal burden and not necessarily in need of protection, care and love [10,11]. However, despite the challenges that they are exposed to, homeless youth are known to be resilient [12,13]. Several factors identified in the literature to foster resilience were categorised in terms of personal resources, interpersonal, environmental and/or socio-cultural factors. 

Personal resources that enable resilience are personal strengths and assets that enable street youth to cope in times of adversity. Such resources identified in the literature include a sense of agency, sense of humour, assertiveness, sense of independence, goals and aspirations, optimism about the future, and unconventional methods such as wearing dirty clothes to elicit pity when seeking help from the public [13,14,15,16,17]. Teasing of friends, although unconventional, is used by street youth to create humour, and as a resource for survival. Three South African studies [14,15,17] showed that street youth generated humour in the context of the street, which promotes survival in adverse conditions. These studies also reported that teasing one another enables street youth not only to shift their focus from the stressors of street life, but also to temporarily forget about their problems. In such situations, humour may thus appear to be used as an adaptable coping mechanism. In a recent study, listening to music and dancing, has also been found to promote resilience [16]. In Malindi’s South African study [16], street youth indicated that the therapeutic use of music and dance may enable street youth to be resilient. This suggests that street youth do not only rely on unconventional means in coping but also have the capacity to use other conventional approaches to cope with the adverse conditions associated with homelessness.

Social relationships and interpersonal resources contribute to resilience among street youth in coping with adversity. In the absence of their immediate family, dependence on friendship, which is created as a model of intimacy and connectedness within the street environment, becomes an important factor for survival [6,18]. In the study of over 70 street working children in Ghana, Mizen and Ofusi-Kusi [18] indicated that friendships formed on the street are based on actions pertaining to food, shelter to sleep, and help during illness. The same study indicated that these friendships are reciprocal and are based on the building of a sympathetic understanding of problems encountered by a street friend and being prepared to help in a meaningful way [18]. Friendships therefore seem critical for survival as it may help a fellow street friend with basic survival such as preventing them from sleeping on an empty stomach, getting a safe place to sleep at night and buying medicine for a sick friend. Some researchers have suggested that this reciprocal friendship only existed when a street youth belongs to a group (usually 3 to 4 people) who share some unique attributes and behaviours [5,7]. This suggests that reciprocal help is strongly influenced by the social network to which one belongs. This is supported by studies conducted in South Africa where belonging to a cohesive group was shown to enhance survival for street children [19,20]. In addition, other studies showed that the ability to access support from friends helps in regulating behaviour on the street. Supportive friendships are valuable to youth to stay away from criminal activities as a result of high social, emotional and financial support that they receive from their fellow street children [7]. On the other hand, it can be argued that adherence to delinquent group values and behaviours might contribute to confrontations with law enforcement officials. 

Other interpersonal factors necessary to foster resilience among street youth include having positive mentors and role models. Studies have demonstrated that these role models are mostly people who have striven for success despite adverse situations in which they live, or previously successful street youth [12,17]. Knowledge of such local or national models serves as ideals to strive for and also in creating a sense of future and hope for these homeless individuals. 

Socio-cultural/community-based factors that have been shown to promote resilience include availability of community-based services, strong faith in God (or a higher being) and strong cultural values and norms [16,21]. According to some researchers [21,22,23], the availability of social services that are usually provided by community-based organisations is necessary in enabling street youth to remain resilient. Some of these services include support from peers, provision of financial help and advice, and the psychosocial services and opportunities offered to homeless youth to develop positive identities, values and social acceptance [12]. These services promote resilience as these support structures, imbedded in the work of community-based organizations, enable these homeless youth access to therapy and counselling advice, personal and technical skills development, as well as in developing a sense of competence. It has also been suggested that reading the Bible by street youth enables them to cope with the stressors of the street because of the positive messages of endurance and hope contained in the Bible [16]. Inspiring religious messages give them hope that their situation will change for the better and make them feel protected and loved at all times [12]. On the street, religious beliefs are strengthened by attending church services, where they are advised and taught to do the “right thing” under all conditions. In Ghana, street children felt that believing and relying on God, and adhering to society rules, would help them overcome the odds in their environment and to eventually succeed [7].

Another emerging area in resilience research is the role of culture in fostering resilience in a particular context [24,25]. Culture comprises collective values, norms, rules and practices unique to a group of people [26]. These rules invariably define and keep such people together [27,28]. Understanding the complexities of culture and how it promotes thriving under vulnerability is crucial as values and practices that foster resilience might vary from one culture to another [25,29]. In a study of young people from both Mexico and South Africa, collectivist cultural values of relatedness and sharing were found to foster resilience [13]. Cultural adherence among other attributes has been identified to be part of the resilience narratives of homeless youth. In a recent South African study among young homeless youth, they indicated that they were proud of their Zulu and Sotho cultures, and that they have learnt a lot from their cultural beliefs and practices, which had enabled them to be resilient [30]. The same study indicated that knowing where one comes from and having adequate knowledge of cultural practices, for example the principles of Ubuntu (humanity towards others), helped foster resilience in street youth [30]. As culture seems to significantly influence resilience among homeless youth [21,22,31], it would be important to examine factors that could foster resilience within a socio-cultural context. Thus, where homeless youth are concerned, such influences include the individual characteristics, connections with peer groups, friends, local community, and society in general and even connections with family.

Within the Ghanaian context, studies have identified reciprocal friendships and having faith as resources for resilience [4,7,18]. However, no study to our knowledge has explored the factors that enable resiliency among street children and youth in Ghana. The main purpose of this study is to examine the factors that foster resilience among street youth in Accra, Ghana. The specific research question was: what are the personal, socio-ecological resilience resources that enable resilience among street youth? The findings are important for understanding the factors and resources that enable homeless youth to withstand the vicissitudes of homelessness.

## 2. Materials and Methods

This study employed a qualitative design with a semi-structured interview schedule, as this allowed the researcher to develop a rich understanding of the lived experiences of homeless street youth and the mechanisms that allow them to survive during adversity. A qualitative approach to research enables researchers to capture how those being interviewed view their world, to learn their terminology and judgments, and to capture the complexities of their perceptions and experiences [32,33]. The sample consisted of 16 children and adolescents (9 males and 7 females) between the ages of 11 and 16 years (average age = 14). Participants were purposively selected from specific locations in the Central Business District of Accra, Ghana based on the following inclusion criteria: self-identified as homeless, lived on the street for a month or more, agreed to participate in the study and willing to answer questions related to their experiences. 

Two social workers who had extensive working experience with homeless young adults through involvement with two Non-Governmental Organizations were recruited to help with the identification of the participants. These research assistants had an average of 8 years’ working experience with street children in various social and psychological domains related to their health and general well-being. Their involvement was to serve as mediators for the researchers to help identify street children at specific locations that met the inclusion criteria and to assure them of the legitimacy of the study as most street youth feel reluctant to open up to people they do not know or trust [4]. The research assistants were, however, not present at the time of interviewing the participants.

The interviews were conducted by the researcher at a convenient place and in a preferred language of the participant (i.e., Twi and Ga—two predominant local languages spoken in Accra, Ghana). Some of the questions asked were: *How do you survive on the street? What would you say are the main challenges for you growing up on the street?; What role does religion play in your life as a young person living on the street?* The interview schedule only served as a guide and probing questions were used to explore the youth’s own views and experiences during the interview process. The interview schedule used for the in-depth interview was developed based on the literature review, the theoretical frameworks and the researchers understanding of the concept of resilience from critical engagement with the literature. Interviews lasted between 45–60 minutes and were transcribed directly into English. 

Ethical approval for the study was obtained from the Department of Social Welfare, Accra, Ghana and the Human and Social Science Ethics Committee of the University of KwaZulu-Natal, South Africa (Protocol number: HSS/1144/012D). Due to their low educational background and inability to read, verbal informed consent was obtained from participants for their participation in the study. The researcher read and translated the consent form into the preferred language of the participant. Permission to audio-record the interview was asked and granted. The social workers were present to act as independent witnesses to the consent process. None of the participants expressed the need for psychological service although they were told of the availability of a psychologist should they require such a service. To ensure confidentiality and anonymity of the participants, the names and location were not reported, as this could lead to identification. 

## 3. Data Analysis

Our analysis followed the six steps involved in thematic analysis [34]. The first step was to translate and transcribe the data verbatim. Because most of the interview was conducted in the local language, the researchers first translated the audio-recorded interview into English. The second stage was the iterative process (where the researchers read and reread the data and delved deep into the data). In the third and fourth stages, the researchers identified themes and codes relevant to the current research aims and objectives. In the fifth stage, identified themes were restructured and revisited to ensure that the analysed data were focused and detailed enough. The final step was where the coded statements were grouped under different broad themes. Two independent coders were engaged to cross-validate the emergent themes. Furthermore, constant engagements with the audio interview were followed to ensure that transcriptions were done properly. Every theme that emerged was discussed with the independent coders and consensus was reached in order to reduce subjectivity on the part of the first author who led the write up. These steps were aimed at enhancing the credibility of the findings. The software, NVivo 10.1 (QSR International, Melbourne, Australia), was used to help structure the analysis patterns. 

## 4. Results and Discussion

In explaining their daily living conditions and survival strategies, it was clear that the youth demonstrated some level of resilience, which enabled them to cope in their newly found environment despite adversity. They derived their strength from (1) intrapersonal resources, which refer to personal characteristics that enhance ways to survive, (2) interpersonal resources, which centred on their interaction with friends, peers and other significant people, and (3) socio-cultural resources available, referring to the cultural influences and community support. These socio-ecological factors identified agree with Ungar’s [35] definition of resilience, which indicated that “in the context of exposure to significant adversity, resilience is both the capacity of individuals to navigate their way to the psychological, social, cultural, and physical resources that sustain their well-being, and their capacity individually and collectively to negotiate for these resources to be provided and experienced in culturally meaningful ways” (p. 225). 

### 4.1. Intrapersonal Resources

For the youth, adapting appropriate coping mechanisms and gaining hope and meaning through their belief in God were essential personal resources for their survival. 

#### 4.1.1. Coping with Humour 

Besides the difficult situations that these street children face on a daily basis in street life, they have adopted some coping strategies that they rely on most of the time. In this study, street children relied mostly on humour to relieve stress and temporarily forgetting about their problems. This was done through teasing and cracking of jokes, as a way to see the bright side of life, and served as an adaptive mechanism to use in their situations of distress. A participant shared his narrative below:

*But my friend will come and crack jokes and I would not cry again. She would come and say something funny, then I would not cry but if not, I can be quiet for a long time*.

(Participant 3)

*It is my friends who come around me and they tell me not to worry. They come around to crack jokes, sometimes some of the funny things we see in the video, they try to act in that way. Then we all begin to laugh. That is what I do to come from my sadness*.

(Participant 4)

The use of humour as a mood enhancement strategy to escape the stress and hassles of the street, as found in this study, has also been reported among street youth in South Africa [16,17,29]. Although teasing may be seen to be socially unacceptable within the general population, in the context of street culture, it promoted mental well-being.

#### 4.1.2. Spirituality

The participants indicated that their belief in an eternal Being (God) helped them to cope and survive on the street. This was manifested in their actions and behaviours on the street. They acknowledged that conditions on the street were very difficult but they believed that God could change things for the better:

*Believing in God gives us hope that things will be good and better one day. I believe in God because whatever He [God] says, he does it. He does not disappoint me*.

(Participant 4)

What is obvious from the narratives above is a strong faith and belief that life can be better. Although they acknowledged their current tough living conditions, they demonstrated here that it would not remain this way forever. Studies have shown that one way in which individuals are able to be resilient during difficult situations is through religion [36]. This strong belief seems to be protective against stress as an individual perceives that help would come during times of crisis. In these situations, individuals conceptualize God as a source of support that can bring stability and relieve distress [37]. Participants in this study had a strong religious belief in God, which was used as a resource for survival. Religion is known to be central to Ghanaians’ life although it’s a secular country [38]. This is clearly reflected in the thoughts and actions of the participants. Religion establishes a moral system that guides an individual’s behaviour, and in Ghana, research has established that religion (as Christianity) exercises a strong regulatory system, helping socialize young people’s behaviour including their sexuality [39,40]. What could have contributed to this could be religious socialization of the Ghanaian child. Although not under the supervision of an adult or a caregiver, such belief in a higher Being (God) does exist and helps regulate the lives of the street youth. Their belief in God was also thought to facilitate access to food and through others by “touching their hearts” to help them when needed:

*God helps me to get food to eat, if not for his grace, I would not get anything when I go to the traffic light to beg. He is the one who touches the hearts of the people to give me money to buy something to eat*.

(Participant 5)

The significance of God in the lives of the street youth is captured in the quotations of the participants below who acknowledge that a higher Being [God] is their protector and provider.

*Some people are even in hospital [sick and not well] and if God has protected me throughout the night, what can’t he do. God can do everything*.

(Participant 1)

*He [God] is the one who takes care of all of us here and if it is not for Him, you will not know what will happen to you. That is why I pray to God that he will help me to be someone good in society*.

(Participant 8)

For these participants, the religious connection they have developed with God, could also be seen as a kind of substituted parental guidance that is non-existent in their lives [41]. For these participants, their religiosity manifested in their personal relationship with God, which they believed played a salient role in their lives. As a result of this, their reliance on a “higher being” is not only to get help in their current predicament but also to maintain an intimate connection with an external being through prayer. Thus, participants had a strong faith and a sense of divine intervention, which has been shown to be associated with resilient people [36,41].

### 4.2. Interpersonal Resources

Apart from the intrapersonal sources of strength mentioned above, the participants talked about reciprocal friendship and engagement in social interactive activities including music and dance.

#### 4.2.1. Friendship

Friendship was viewed as being important to the youth’s survival on the street. Participants in this study received help from friends with the understanding that they could return such a favour in the near future. These friendships are based on receiving and giving of food, sharing of clothes and providing drugs in times of ill health. This was revealed in the narratives of the majority of the participants:

*I only get help from my friends. A close friend can get you some money to buy food, and then you can give the money back [to the person] when you get it. A good friend can also share his food with you. The last time, my friend was not having any food to eat, I called him to come and share my food with me*.

(Participant 14)

*It’s your friend who will give you some food that is if she has enough. If they don’t give me [some food] when I am in need, I will also not give them when they ask me*.

(Participant 3)

Friendships as found in this study provide street children with a sense of family and intimacy, a finding which is consistent with the assertion that supported relationships foster resilience among individuals in adversity [12,20]. It has been reported that South African street youth reciprocated and helped their friends and encouraged them to seek and search for other available resources [12]. Therefore, friendship may offer some form of security and emotional attachment for homeless youth in Ghana.

Sharing important basic needs such as food with friends is done in anticipation of receiving a favour in exchange. Friendships developed based on reciprocity thrive when the people involved understand the problems and are willing to act in meaningful ways. This form of street living arrangement, that has been described as “*shared living*” among street children, provides a strong sense of reciprocal living under extreme conditions [12,18]. This phenomenon has also been described as redistributive exchange, where street children in Port-au Prince bring their resources together in order to guarantee daily meals for all [42]. Although this was not observed among the street children in this study, giving of money and food with the intent of receiving the favour back may have saved other street children from going hungry or being without food for days.

It was also observed that the majority of youth living on the street walked in a group of 2 to 3 people, and that sharing of resources was based on the relationship in the group. In remarking that, “*If you are not my friend, I would not borrow you my dress*” (Participant 15) and “*I only give to my friend that I walk with, and sometimes with those who stays with me always*” (Participant 3), participants indicated that reciprocating favours among them is greatly influenced by the social network to which you belong. This clearly shows the importance of belonging to a group or having close ties with friends whom you identify with. Peer group support has also been reported among South African street youth who reciprocate and help their friends and encourage them to seek and search for other available resources [12]. These living arrangements where street children live in a cohesive group and help those with whom they are closely ‘connected to’ may offer some form of security and emotional attachment [19]. 

#### 4.2.2. Social Interactive Activities 

The majority of the youth engaged in social interacting activities, which helped them to cope. These interactive activities included sporting activities and friendships. These activities encouraged and protected them from risky behaviours and enhanced their survival on the street. The majority of the male participants indicated that playing football was one of the main activities that they really liked doing during the day. This was expressed in their narratives:

*Sometimes when we are not doing anything, we group ourselves into 2 and play football with each other. We do it as if there is some trophy to be won [laughs] because you do not want to find yourself in the losing team*.

(Participant 16)

The narratives above portray street youth as social actors who actively developed ways of coping. Their involvement in activities such as listening to music and dancing, as well as participating in sports enabled them to cope and to some extent forget about the problems they faced in their daily lives. This reflects adaptability, which has been argued as an attribute of resilient individuals in adversity [43,44]. The process of adaptability was evident in this study as the participants showed signs of being particularly resilient [45]. An individual’s ability to make meaning from a new environment is influenced by the coping mechanism adopted, the problems faced and the availability of resources within one’s environment [22,31,46]. Coping can be defined as changing one’s cognitive and behavioural efforts to manage specific internal and/or external demands that are perceived as exceeding one’s personal resources [47]. This therefore presupposes that the types of coping strategies adopted by individuals to deal with daily problems or hassles are contextually driven.

### 4.3. Socio-cultural Resources 

Socio-cultural factors that enhance survival of the homeless children were divided into two categories: cultural norms and community-based support (e.g., help from NGOs and other community members). Similar to findings in previous studies among youth conducted elsewhere, cultural norms and community-based support systems were identified as important environmental factors that enhance resilience [48,49,50].

#### 4.3.1. Cultural Norms

The term cultural norm is used in this context to refer to stories and advice received by participants from their parents and or relatives before moving to the streets. These values were adhered to and included in their daily lives and seemed to regulate and guide their behaviours in times of difficulties. Some participants shared their views in this regard:

*I overcome challenges here [on the street] by remembering what my father and mother told me. He [my father] told me not to be sad, and that even if he died, I should not be sad. I said thank you to him. I told him not to say that, but after another one week, he was sick again, and died. My mother also told me that I should not be sad because God was with me and He will take care of me. For the Lord who created me will protect and take care of me*.

(Participant 1)

This narrative of the participant (an orphan who lost both parents before her 9th birthday) indicated how much she relied on the advice given to her by her parents, and that her ability to cope was as a result of the adherence and reflections on these values. The internalization of such wisdom into their moral values helped regulate their behaviours irrespective of where they lived. A violation of these values or the inability to live accordingly created feelings of sadness and a sense of regret. This finding supports the study of Theron et al. [30] who indicated that resilience among Black South African youth is strengthened by living in a community that accepts, supports and encourages cultural traditional practices and beliefs. Other researchers such as [50] have suggested that for at-risk youth who have considerable knowledge of their cultural heritage, adherence to such cultural rites can facilitate positive identification with a larger social grouping, along with its associated protective process of cultural pride. 

Ghanaian street youth in this study, who used such internalized values as resources, had benefited from a strong cultural background of intense interaction with a close family relative (either father or grandparents). This history encouraged them to uphold such values and norms at all times irrespective of the situation. We can therefore imply that cultural values helped promote resilience. However, as culture has been shown to be fluid it can be implied that culture may not be operating as a protective mechanism all the time [48]. Accessibility to community-based services that provide training and fulfil the physical needs of homeless youth plays a significant role in their survival and adaptation as previously reported by Ghanaian researchers [4,7]. 

#### 4.3.2. Community-based Care and Support 

Participants reported that they were receiving enormous support and help from different people within the community. Being homeless and living in adverse conditions, gave them a chance to accept help from other individuals and organizations. The majority of the participants indicated that they had visited and received help from a community-based NGO working with children and youth living on the street:

*I have been to CAS (Catholic Action for Street Children). They give us books and pens, and then give us class work to do. When we go there in the morning, they give us water to bath and give us some clothes to wear. They will give us money to buy porridge for breakfast. After 12:00 noon, we will get our lunch, and then we leave there to our various places at 4:00pm in the afternoon*.

(Participant 4)

*I have been to the Catholic Action for Street Children [CAS]. They train us and they advise us to live well. I know that if I follow the advice they give us I know it would help me in my life, but the problem is, most of us do not follow the advice. Some of the girls listened to them are now doing their own work (having jobs). We go there in the morning to learn a trade [apprenticeship], and when they see that you are OK, they will take you to a place in Ashiaman [a suburb in Tema] where you will learn more. When they think that you are now ready, then they will open a shop for you to be on your own*.

(Participant 13)

The above mentioned NGO does not only provide for the physical basic needs of these street youth, but also provide them with some skills training, including life skills, with the view of youth becoming economically independent adults in the future. A strong belief was explained by Participant 4 that they could succeed if they followed the advice given to them as some previous street girls who were willing to be trained, had done. The instructions given to them through training had helped the former street girl to succeed and to become independent. In the absence of governmental funded organizations mandated to take care of homeless youth in Ghana, these community-based organizations provide homeless youth with opportunities to receive various services that are likely to help alleviate the adverse health and well-being effects resulting from living on the street. 

The findings of this study have implications for multilevel prevention interventions based on the ecological systemic framework, with emphasis on personal, interpersonal and community based levels are needed. Such programmes should engage youth as early as possible when they become homeless to decrease the degree of deterioration in physical and mental health, as prolonged periods of time on the street leads to have higher psychological distress and lower reported resiliency. First, at the individual level, there is a need for programmes aimed at building resilience in youth to cope better with the stressors of living on the streets by enhancing psychological resources of homes youth. These individuals could be provided with some skills training including life skills, with the view of youth becoming economically independent adults in the future. Secondly, fostering of health enhancing social networks that provide homeless youth with an alternative network for gaining social support to gangs, which promote anti-social behavior, should be considered. The positive influence of role models, especially homeless youth who have been successful and had overcome the adverse conditions of street life, could provide advice and form support systems for those on the street especially in terms of how to deal with issues of drugs, and how to beat the odds of the street. Finally, at the community and societal levels, the role of NGOs and the services and programmes they provide for such a vulnerable population are very crucial. Such programmes should aim at re-integrating youth in social life could utilize these specialized skills that the street youth acquired, in their training and educational programmes to the youth advantage.

## 5. Strengths and Limitations

The findings of this study make at least two unique contributions to the literature in the field, as it extends the findings of a previous study conducted in Ghana [18], which indicated that reciprocal friendship-asking, giving and receiving served as protective factors for survival for homeless street children. In addition to this mechanism, this study has identified that strong religious beliefs, reliance on internalized cultural values and norms could serve as protective factors and build resilience among street youth, which in turn enhance their survival. Mizen and Ofosu-Kusi [18] clearly indicated that an interpersonal resource (strong friendship), could promote survival, but the study could not explicate the extent to which other intrapersonal and community-based resources served as resources for survival. The findings of this study have to some extent delineated and explicated certain additional intrapersonal mechanisms that promote resilience such as strong religious beliefs, and adherence to internalized cultural values and norms. Despite this strong contribution the small sample size and the non-probability sampling method used mean that the findings cannot be generalised to all street children in Ghana. The sample used in this study were only street youth who lived entirely on the street, and as such the experiences of those in the shelter could have yielded another understanding of resilience. Further studies should therefore include street youth who are housed in the various shelters provided by community-based organizations in Ghana as there are NGOs that provide education and reproductive health services to homeless children in Accra. Their views could validate and provide further understanding of the general risk and resilience factors for homeless youth in Ghana. Despite these shortcomings, the findings in this study make significant contributions to the area of resilience among street youth in a Ghanaian context and beyond.

## 6. Conclusions

The current study, which was conducted to explore factors that foster resilience among street youth in Accra, has revealed many significant findings. The findings showed street youth to be social actors who actively develop ways of coping as they benefited from three main type of sources: intrapersonal resources (coping with humour and religious beliefs), interpersonal resources (reciprocal friendships and cohesive group living) and socio-cultural resources (cultural norms and community-based care and support structures). These identified important protective factors could be strengthened in the development of mental health interventions to help reduce the impact of adverse conditions that are likely to impact the young lives of street youth. While these resources seem to ‘reside’ at three different levels (intra/interpersonal & social), in the dynamic of street (and in general) life, they overlap and interact. The belief in a higher power both directs and explains social interactions (giving behavior, etc.), as interpersonal norms derive from and inform such interactions too, primarily inside-group ones. It appears as though they have built a mini-society that is governed by parallel yet similar rules to the larger one. Furthermore, understanding the factors and resources that enable homeless youth to withstand the vicissitudes of homelessness, we should strive to provide alternative solutions to living on the streets.
